# Supplementary material for: SyNDI: synchronous network data integration framework
Source: BMC Bioinformatics. 2018 Nov 6;19:403. doi: 10.1186/s12859-018-2426-5 (PMC6219086; doi:10.1186/s12859-018-2426-5)
Supplement: Supplementary file 5 — Files of the example presented in Additional file 4. This additional file is a zip package that contains all files used in Additional file 4. Detailed descriptions of each of these files are presented in a README file included in this zip package. (GZ 30411 kb) [file 12859_2018_2426_MOESM5_ESM.gz › SyNDITools/Meme2Fimo/meme_4.9.0_4.tar.gz!/meme_4.9.0/doc/clustalw2fasta.html]

clustalw2fasta


# clustalw2fasta

**Usage:** `clustalw2fasta [options] <alignment
file>`

**Description:**

This program converts a multiple alignment in clustalw
format into a FASTA file.

**Input:**

- <*clustalw file*> - a file containing one or more
  aligned sequences in Clustalw format.

**Output:**

The program prints to standard output each of the sequences in the
multiple alignment, in FASTA format.

**Options:**

- -consensus <*threshold*> - Rather than printing each of
  the sequences to the FASTA file, the program prints their consensus,
  with gaps (hyphens) inserted whenever the consensus character occurs
  in less than <*threshold*>% of the sequences.
- -nogap - Do not include gap characters in the output.
- -seqorder <*seqorder filename*> - Output the sequences in the order
  given in the <*seqorder filename*> file. This file must contain
  a superset of the sequences in the Clustalw alignment file.

**Bugs:** None known.
